# Self-Identity and Career Success of Nurses in Infectious Disease Department: The Chain-Mediating Effects of Cognitive Emotion Regulation and Social Support

**DOI:** 10.3389/fpsyg.2020.563558

**Published:** 2020-11-27

**Authors:** Chao Wu, Shuang Li, Feixia Cheng, Linyuan Zhang, Yanling Du, Shizhe He, Hongjuan Lang

**Affiliations:** ^1^Nursing Department of Air Force Medical University, Shaanxi, China; ^2^Tangdu Hospital, The Second Affiliated Hospital of Air Force Military Medical University Shaanxi, Xi’an, China; ^3^Foreign Training Group of Naval University of Engineering, Hubei, China

**Keywords:** infectious diseases department, nurses, self-identity, cognitive emotion regulation, social support, chain-mediating effects, structural equation modeling

## Abstract

There has been some research conducted regarding nurses’ career success aimed at exploring its influencing factors, but there is no research on the mechanism of self-identity on the career success of infection control nurses. In order to further explore the formation mechanism of career success of nurses, we conducted our study using the Kaleidoscope Career Model to explore the chain-mediating effects of cognitive emotion regulation and social support between self-identity and career success. Five hundred forty-seven infection control nurses from nine different hospitals participated in the study and completed questionnaires on the self-identity, cognitive emotion regulation, social support, and career success scales. The results from structural equation modeling reveal that self-identity has a positive effect on positive emotion regulation and social support and a negative impact on negative emotion regulation. Positive (negative) emotion regulation has a positive (negative) effect on social support. All antecedents have a positive impact on career success except negative emotion regulation, which has a negative one. Bootstrap analysis shows that the relationship between self-identity and career success is partially mediated by the chain of cognitive emotion regulation and social support. Overall, our research sheds light on the mechanism of self-identity on career success of nurses. Theoretical and practical implications are discussed.

## Introduction

Modern society is widely intertwined, but the development of the transportation industry and subsequent economic and cultural exchanges helped speed up the spread of diseases (Lee et al., 2019). Nowadays, as different strains of infectious diseases emerge with their own resistance profiles, medical professionals face unprecedented challenges (Musau et al., 2015). As a special profession, infection control nurses who specialize in preventing and treating infectious diseases need to master more medical knowledge and strict operating procedures for self-protection than other nurses (Stirling et al., 2004). They not only treat infectious patients, but also predict infectious diseases and report to the public in a timely manner (El-Bahnasawy et al., 2014; Wood et al., 2019). They play an important role in infectious disease work, and improving their career success is a significant issue. A sense of career success is the realization of an individual’s positive psychological satisfaction and self value, which is conducive to the improvement of work quality and efficiency (Robinson et al., 2016; Cumbler et al., 2018). Therefore, improving the career success of infectious disease nurses is of great importance and is helpful to promote the development of infectious disease nursing and public health.

Career success refers to both the positive psychological feelings that individuals gradually accumulate and acquire in their work experience and work-related achievements (Zamanzadeh et al., 2019; Xin et al., 2020). Achieving career success is defined as developing individual potential, meeting the satisfaction of growth needs, and realizing individual self-value (Woolston, 2019). Previous research on the career success of nurses mainly focused on either objective factors (Dan et al., 2018) or internally and externally driven factors (Wang et al., 2019). Among these, self-identity is an important psychological factor actively affecting career success (Arrowsmith et al., 2016). The sense of self-identity is a person’s degree of self-cognition, which is a synthesis of individual inner feelings, self-consciousness, and external evaluation (Meijer et al., 2018). Scholars and practitioners have increasingly urged the need for nurses to strengthen self-identity in recent years (Brown et al., 2006; Tai, 2011; ten Hoeve et al., 2014; Peterson, 2016). It is an important factor in achieving career success (Dan et al., 2018) in that individuals with good self-identity have clear life goals, experience self-worth, and gain either social recognition or approval in the process of pursuing their goals (Werner and Hochman, 2019). They are always energetic and active, which is conducive to the completion of work tasks and the realization of work objectives (Li et al., 2012).

According to Loeffler et al. (2019), the concept of emotion regulation can be regarded as a cognitive way to manage the intake of emotionally arousing information that has an impact on the higher cognitive activities of reasoning, decision making, and so on. Emerging research suggests that the development of self-identity is also closely related to cognitive emotion regulation in that good self-identity can help individuals choose positive emotion regulation strategies and avoid negative ones (Dereboy et al., 2018; Southward and Cheavens, 2018). Social support refers to the help that individuals get from the outside (Hu et al., 2018). Indeed, previous research has reported that self-identity is also related to social support (Youngkeun, 2020) in that individuals with a good sense of self-identity often have better psychological makeup and harmonious interpersonal relationships, which is conducive to mobilizing the available resources around them (Werner and Hochman, 2019), are they are more likely to seek help from outside. An 8-month study of nurses in Australia showed that self-identity was effective in predicting social support through work (Merrick et al., 2012).

Cognitive emotion regulation is closely related to social support. Positive emotion regulation strategies can promote good psychological adaptability and positive coping and actively seeking help from the outside world (Bamonti et al., 2019; Young et al., 2019), which is consistent with d’Arbeloff’s research (d’Arbeloff et al., 2018) that cognitive emotion regulation is significantly related to social support, and good social support can successfully regulate negative cognitive emotion. Good self-identity can regulate the cognitive emotion strategy (Sharma et al., 2018), and individuals with good self-identity tend to choose the positive cognitive emotion strategy, which is helpful to get help and social support from outside. Social support is an important factor for improving the psychosocial work environment and fostering a positive work climate and is conducive to the acquisition of career achievement (Johnsen et al., 2018; Wang et al., 2018). A good social support system can help nurses to prevent the onset of work-related stress, resolve work difficulties, and provide career planning, which is an important factor for nurses’ career development and success (Al-Hussami et al., 2018; Southward and Cheavens, 2018).

The Kaleidoscope Career Model (KCM) was first applied to the study of female career success and analysis of the influencing factors of career success in 2018 (Margie and Iin, 2018). The application scope of KCM expanded as researchers conducted more studies (Eileen et al., 2019). It asserts that career success is mainly composed of three factors: authenticity, challenge, and balance (Lisa et al., 2018): (1) Authenticity is the parameter that describes being genuine and true to oneself, knowing one’s strengths and limitations, having a correct examination of oneself. In our study, we used self-identity to reflect this parameter. (2) Challenge as a driving force is an indispensable factor in career development. Individuals who focus on challenge often hold themselves to a higher standard. Taking different measures to deal with challenges or difficulties may be a greater challenge for individuals and can have an important impact on career success. In our study, it is a challenge for nurses to choose different cognitive emotion regulation strategies in clinical work. (3) The balance parameter refers to the balance between an individual and the outside world, including colleagues, family, friends, etc. Good social support is helpful for individuals to achieve this balance in the process of career success. Our study, which is based on KCM, aims at exploring the career success of infection control nurses in a more efficient manner.

There must be some research that are conducted regarding nurses’ career success and aiming at exploring its influencing factors, but there is insufficient discussion on the mechanism of self-identity on career success of infection control nurses. Therefore, it is necessary to study the self-identity of infection control nurses and its impact on career success. On the basis of these studies, we applied KCM to analyze three factors: authenticity (self-identity), challenge (cognitive emotion regulation), and balance (social support) and hypothesized the following: (1) Nurses’ self-identity will affect cognitive emotion regulation. (2) Positive emotion regulation is conducive to seeking social support and further promotes career success. (3) Negative emotion regulation is not conducive to seeking social support and further hinders career success. (4) Cognitive emotion regulation and social support play a chain-mediating role between self-identity and career success. The goal of this study is to further enrich the mechanism of self-identity on career success and to provide a theoretical basis for strengthening nurses’ self-identity and promoting career success to improve the quality of nursing.

## Materials and Methods

### Participants and Procedure

Participants were from infectious disease departments of nine hospitals in China. With the help of the hospital managers, surveys were randomly sent to 583 infection control nurses from January to July 2020 using a convenient sampling method. The inclusion criteria were nurses in infectious disease departments who had obtained professional registration and agreed to participate in the study. The exclusion criteria were those who were not willing to participate or were absent during the survey. Prior to conducting the study, written and informed consent were obtained from the participants. The time of filling in the questionnaire was controlled between 15 and 30 min. After the nurses completed the self-fulfilled questionnaire via a paper and pencil process, the researchers immediately collected it. In the process of filling in the questionnaire, 13 nurses quit the survey and 23 questionnaires were found to be incomplete. After collecting the questionnaires, 547 questionnaires (93.83%) were determined to be valid.

### Ethics Statement

This study was conducted under ethical guidelines described in the Helsinki Declaration (World Medical Association, 2013). Ethics approval was not required because there was no unethical behaviors in the study and our study did not involve human clinical trials or animal experiments. Before the investigation, we explained the purpose to the participants, asked for their verbal consent before conducting the survey, and had them sign the informed consent form. During the investigation, participants could terminate and withdraw from the investigation at any time, and the questionnaire was completed anonymously.

### Measures

#### Self-identity Scale

Self-identity was measured by the Self-identity Scale (SIS), which was compiled by psychologists Ochse and Plug according to Eriksson’s theory (Ochse and Plug, 1986). The scale has been translated and debugged into a Chinese version by scholars and has been widely used in China with good reliability and validity (Qasim et al., 2019; Wu et al., 2019). There are 19 items on the scale, such as “I am proud of being a member of the group” and “my value is recognized by others.” Participants rate the items using a 4-point Likert scale (1 = strongly disagree; 4 = strongly agree). Reverse questions are converted to reverse scoring. The sum of all the questions is the total score of the questionnaire. Higher scores indicate better development of self-identity. In this study, the Cronbach’s alpha coefficient for the SIS results was 0.831.

#### Cognitive Emotion Regulation Questionnaire

Cognitive Emotion Regulation Questionnaire (CERQ) was developed by Garnefski et al. (2017). The scale quantifies the coping strategies of individuals when they are faced with events beyond their own capacity. Previous scholars have translated and debugged this scale into a Chinese version widely used in China (Tan et al., 2018; Wu et al., 2018; Zhang et al., 2019). There are 36 items on the scale, including nine dimensions: acceptance, active refocus, rational analysis, active reevaluation, active replanning, self-blame, other blame, disaster, and meditation, using phrases such as “I look for positive aspects of things” and “I think these mistakes are caused by others.” Among the nine dimensions, the first five belong to adaptive strategies, and the last four belong to non-adaptive strategies. The scale’s scoring system was 1–5, with 1 meaning never and 5 being always. The higher a participant’s score, the more likely the individual is to use this specific cognitive regulation strategy. The overall Cronbach’s alpha coefficient of the scale was 0.747, and the Cronbach’s alpha coefficient of each dimension was between 0.719 and 0.868 in our study.

#### Social Support Rating Scale

The Social Support Rating Scale (SSRS) was compiled by Chinese scholar Xiao Shuiyuan in 1986. It has since been adopted by many scholars in China, showing good reliability and validity (Xiao et al., 2020). Social support refers to material conditions and resources as well as emotional support. There are 10 items on the scale, including three dimensions: objective support (three items), subjective support (four items), and utilization of social support (three items). For example, “Which way do you choose when you are in trouble?” and “How many close friends do you have?” The total score of the scale reflects the status of individual social support. The Cronbach’s alpha coefficient of this study was 0.761, and the Cronbach’s alpha coefficient of the dimensions 0.721, 0.742, and 0.837.

#### Career Success Scale

Career success refers to the individual’s positive psychological feelings accumulated and obtained in the work as well as their work achievements (Li et al., 2014). The scale has two dimensions and 11 items. There are six items in the dimension of professional competitiveness and five items in the dimension of professional satisfaction in Career Success Scale (CSS). Examples of professional satisfaction statements include “the company thinks I am very useful” and “I have many development opportunities in the company.” The scale uses a 5-point scoring method with 1 being highly disagree to 5 being highly agree, respectively. The scale has been widely used and has good reliability and validity. The Cronbach’s alpha coefficient of this study was 0.765, and the Cronbach’s alpha coefficients of the dimension of professional competitiveness and professional satisfaction were 0.721 and 0.765 in the study.

### Data Analyses

First, we used exploratory factor analysis to test the possible common method bias (Wingate et al., 2018). We sorted out each item of the questionnaire and used SPSS 21.0 for exploratory factor analysis. In this study, we found that the first common factor interpretation rate was 30.23%, which was less than the critical standard of 40%. This showed the common method bias could not be a concern in this study. Then, we analyzed the descriptive statistics of each variable and used the Pearson correlation coefficient to analyze the correlations among self-identity, cognitive emotion regulation, social support, and career success. Finally, a two-step procedure of structural equation modeling (SEM) was adopted to analyze the chain-mediating effects of cognitive emotion regulation and social support between self-identity and career success (Li, 2011). Specifically, the measurement and structural models were performed using Mplus 8.0 in two sequencing steps to examine our hypotheses. Then, we ran 5,000 bootstrapping resamples to examine the multiple mediator effect. The 95% confidence interval does not contain 0, which signifies statistical significance (Shrout and Bolger, 2002).

## Results

### Descriptive Statistics

We conducted descriptive and correlation analysis on the data, and the analytical results are shown in Table 1. The results show that self-identity is positively correlated with positive cognitive emotion regulation (*r* = 0.228, *p* < 0.01), social support (*r* = 0.496, *p* < 0.01), and career success (*r* = 0.402, *p* < 0.01). Negative cognitive emotion regulation is negatively correlated with self-identity (*r* = −0.471, *p* < 0.01), social support (*r* = −0.334, *p* < 0.01), and career success (*r* = −0.181, *p* < 0.01).

**TABLE 1:** Means, SD, and inter-correlations.

	1	2	3	4	5
1. Self-identity	1				
2. Positive CER	0.228**	1			
3. Negative CER	−0.471**	0.017	1		
4. Social support	0.496**	0.245**	−0.334**	1	
5. Career success	0.402**	0.217**	−0.181**	0.363**	1
*M*	61.07	67.12	40.75	34.43	41.14
SD	6.37	7.07	6.13	5.39	5.03

N = 547. **P < 0.01, all tests were two-tailed. CER, cognitive emotion regulation.

### The Mediation Model

We use confirmatory factor analysis to test whether the fitting index of the measurement model conformed to the requirements. The results show that the fit indexes are χ^2^ = 412.39, df = 110, χ^2^/df = 3.75, Tucker-Lewis index (TLI) = 0.906, comparative fit index (CFI) = 0.924, root mean square error of approximation (RMSEA) = 0.071, and standardized root mean square residual (SRMR) = 0.075.

Structural equation modeling has a sample size requirement. When the number of entries is too large, more parameters need to be estimated by SEM. If the sample size is small, large parameter bias may be produced by using the original items. Item parceling can improve the communalities and reduce random error, which is an effective method to solve this problem (McDonald and Ho, 2002). We packed 19 items of SIS into three packages according to the item packing method of high to low strategies (Cho and Kang, 2007). For the other three scales, we packaged them according to their respective dimensions. Then, we tested whether the fitted index of the constructed structural equation model conformed to the requirements. The results show that the fit indexes are χ^2^ = 412.39, df = 110, χ^2^/df = 3.75, TLI = 0.906, CFI = 0.924, RMSEA = 0.071, and SRMR = 0.075. Figure 2 depicts the mediating effect model. Self-identity had a significant positive impact on positive cognitive emotion regulation (β = 0.129, SE = 6.588, *p* = 0.000) and indirectly affected social support (β = 0.392, SE = 3.661, *p* = 0.000) and career success (β = 0.363, SE = 3.310, *p* = 0.001). Self-identity had a significant negative impact on negative cognitive emotion regulation (β = −0.290, SE = −9.226, *p* = 0.000) and indirectly affects social support (β = −0.236, SE = −2.478, *p* < 0.05) and career success (β = 0.363, SE = 3.310, *p* = 0.001). Self-identity was positively related to career success (β = 0.251, SE = 2.736, *p* < 0.01). The total indirect effect accounted for 44.28% of the total effect of self-identity on career success (total effect = 0.542, total indirect effect = 0.240, *R*^2^ = 0.394).

**FIGURE 1:**
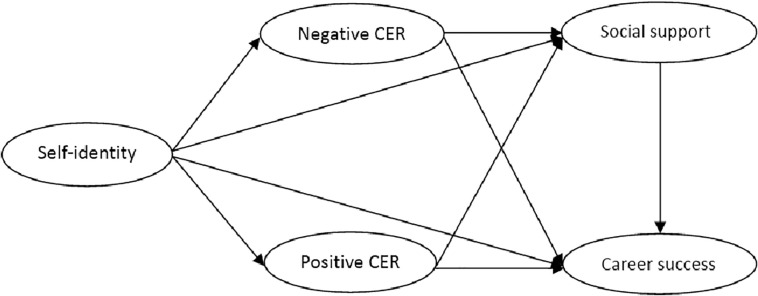
The multiple mediator model of self-identity and career success hypothesized: (1) Nurses’ self-identity will affect cognitive emotion regulation. (2) Positive emotion regulation is conductive to seeking social support and further promoting career success. (3) Negative emotion regulation is not conductive to seeking social support and further hindering career success. (4) Cognitive emotion regulation and social support play a chain mediating role between self-identity and career success.

Table 2 shows the confidence interval of mediating effect value in the chain-mediated model. The results show that the path from self-identity to career success through social support is significant. Confidence intervals are 95% (0.066, 0.272), which did not contain 0, indicating that the chain-mediating effects were significant. The path from self-identity to career success through the chains of positive/negative cognitive emotion regulation and social support are significant. Confidence intervals are 95% (0.001, 0.049) and (0.002, 0.035). However, the other paths are not significant. The total mediating effect value was 0.199, *p* < 0.01, and the 95% CI (0.073, 0.326) did not contain 0, indicating that the total mediating effect was significant. The specific path model diagram is shown in Figure 1.

**TABLE 2:** Confidence interval of mediating effect value in chain mediated model (5,000 bootstrap samples).

Model path	Estimate	95% CI	
		LLCI	ULCI
SI → SS → CS	0.169	0.066	0.272
SI → PCER → SS → CS	0.025	0.001	0.049
SI → NCER → SS → CS	0.018	0.002	0.035

## Discussion

The present study investigated the influence of self-identity on career success of Chinese infection control nurses, and we selected cognitive emotion regulation and social support as mediating variables to investigate this effect. The results suggest that self-identity has a direct positive impact on career success of nurses and indirectly through cognitive emotion regulation and social support. Cognitive emotion regulation and social support as a chain structure mediate the relationship between self-identity and career success. The size of the total mediating effect is 44.28%, which indicates that the constructed hypothetical model has a certain explanatory power to describe the influence of Chinese infection control nurses’ self-identity. The model features three significant paths, which are from self-identity to career success through the mediating effect of social support and the chain of positive/negative cognitive emotion regulation and social support, signifying that both social support and social support linked together with cognitive emotion regulation play an important mediating role.

**FIGURE 2:**
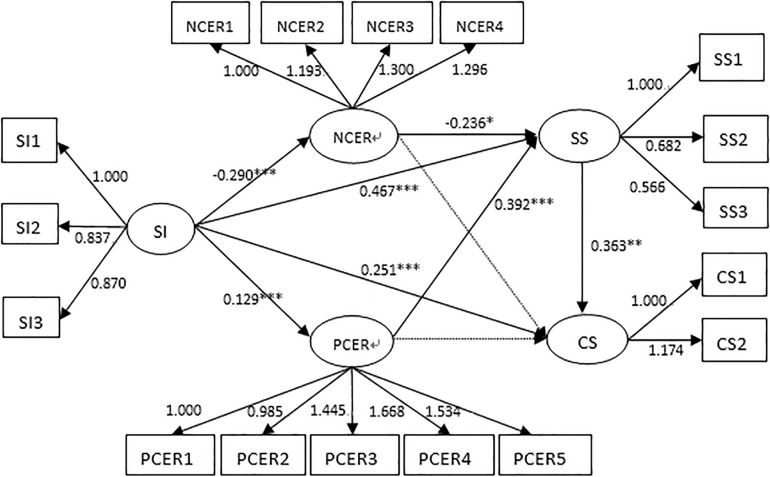
The multiple mediator model of self-identity and career success. SI, self-identity; SI1–SI3, manifest variables of self-identity; NCER, negative cognitive emotion regulation; NCER1–NCER4, manifest variables of negative cognitive emotion regulation; PCER, positive cognitive emotion regulation; PCER1–PCER4, manifest variables of positive cognitive emotion regulation; SS, Social support; SS1–SS3, manifest variables of negative cognitive emotion regulation social support; CS, career success; CS1–CS2, manifest variables of career success; ^∗^*p* < 0.05, ^∗∗^*p* < 0.01, and ^∗∗∗^*p* < 0.001.

Positive self-identity can help individuals properly understand and accept themselves and make an individual energetic, not immersed in lament or complaint (Sokol and Serper, 2017). Nurses with good self-identity have a clearer goal and could better solve their difficulties in clinical work, which will result in a greater chance of career success (Kern et al., 2009). Social support plays a good buffer role in a person’s psychological stress process (Liu et al., 2016). It includes objective forms, such as material direct assistance and subjective forms of an individual’s emotional experience and satisfaction with how they are respected, supported, and understood (Liu et al., 2016; Manning et al., 2020). Good social support is conducive to the formation of a better interpersonal network and the improvement of individual work efficiency and engagement and the solution of work problems (Rojas-Guyler et al., 2007). In Figure 2, the SEM shows that self-identity has a positive (negative) effect on positive (negative) emotion regulation, which has a positive and negative effect on social support. Cognitive emotion regulation refers to the cognitive coping strategies taken by individuals in dealing with events (Qiao et al., 2016; Loechner et al., 2020). Our study is consistent with the results of Cai et al. (2017) who find that positive (negative) cognitive emotion regulation is positively (negatively) correlated with social support. When individuals adopt positive emotion regulation strategies, they seek help from the outside world by communicating with others to find solutions to their problems (Cano et al., 2020; Loechner et al., 2020). When individuals adopt negative emotion regulation strategies, they experience self-closure, which prevents them from seeking out social support. The chain intermediary model proves that, when individuals have a high sense of self-identity, they view themselves positively and always adopt positive cognitive emotion regulation strategies to seek help and social support when they encounter negative events; they are more likely to experience career success while negative cognitive emotion regulation is not conducive to social support and plays a negative predictive role in career success.

Infection control nurses regularly face dangerous and infectious diseases, high risk of occupational exposure, and risk of infection (Jin et al., 2020). The demanding nature of infection control work makes these nurses experience greater psychological pressure (Qiao et al., 2016). The significance of an individual’s career success helps them to realize its value and play a corresponding role in the post (Robinson et al., 2016). Compared to other nurses, they should have better self-identity, believe that they can be competent for the post responsibilities, handle the work well, and do a good job in disease care. Therefore, to study their career success is of great significance to improve the quality of nursing. Nurses and managers should be aware of the chain-mediating role of cognitive emotion regulation and social support between self-identity and professional success, so they can apply it to their professional work. Nurses can learn how to improve self-identity and regulate their emotions through theoretical teaching, and managers can maintain open lines of communication with the nurses to ensure they are emotionally and professionally supported. When infection control nurses encounter negative events in their work, they should learn to seek help from colleagues, leaders, or family members. They can do this by adopting positive cognitive and emotional adjustment strategies. The director of the hospital should pay attention to the self-identity of nurses and give proper encouragement and should also strengthen the support to the nurses, both psychologically and materially. Material support, such as bonus and reward, will mobilize the enthusiasm of the nurses and subsequently promote their professional success. Therefore, their hospitals, family, and friends should also give support and care to infection control nurses.

### Implications for Researchers and Practitioners

This study is of great significance to researchers, nurses, and hospital managers. The infection control nurses should strengthen their self-identity and learn to adopt a positive way of cognitive emotion regulation when dealing with problems. This will result in nurses promoting harmonious relationships with their leaders, colleagues, family members, and friends, which is ultimately conducive to better career success. The infection control nursing managers should support the professional development of their nurses by providing social support and giving them the tools needed to build good self-identity and positive emotion regulation strategies.

### Limitations

There are some limitations in this study. Because the questionnaire was self-report, it was subjective and would produce bias. Only 547 infection control nurses in China were fully surveyed, leading to a sample size that was not large enough, and the region has limitations. There are omitted variables known to influence career success, such as organizational ones (Chen et al., 2012; Kowalczuk and Krajewska-Kułak, 2015; Wang et al., 2018), which we had not studied in our research. If this issue were included in study, it would have a positive influence on career success. Our results can be relevant out of a local context and in both health professionals and non-health professionals in theory, but we have only investigated infectious disease nurses actually, in that this group has its particularity. It is of great significance to study their career success in promoting the development of infectious disease nursing. However, it is not clear whether it is applicable to non-health professionals in practice. We hope that, in future research, we can further expand the sample size, collect larger amounts of data in different professions and different regions, and discuss the organizational factors or other factors in our study.

## Conclusion

We enrich the mechanism research on career success. Through the exploration of the career success of infection control nurses in our study, it is shown that self-identity has a positive predictive effect on career success, and the chain-mediating effects of cognitive emotion regulation and social support are significant between self-identity and career success.

## Data Availability Statement

The raw data supporting the conclusions of this article will be made available by the authors, without undue reservation.

## Ethics Statement

Ethical review and approval was not required for the study on human participants in accordance with the local legislation and institutional requirements. The patients/participants provided their written informed consent to participate in this study.

## Author Contributions

All of the authors contributed to the design of the study, the distribution of questionnaires, the collection of data, the writing of manuscript, and the submission of contributions.

## Conflict of Interest

The authors declare that the research was conducted in the absence of any commercial or financial relationships that could be construed as a potential conflict of interest.

## Abbreviations

SIS: Self-identity Scale
CERQ: Cognitive Emotion Regulation Questionnaire
SSRS: Social Support Rate Scale
CSS: Career Success Scale
KCM: Kaleidoscope Career Model
SEM: structural equation modeling.
